# Association between triglyceride glucose index and sleep disorders: results from the NHANES 2005–2008

**DOI:** 10.1186/s12888-022-04434-9

**Published:** 2023-03-10

**Authors:** Heng Pei, Shuyu Li, Xin Su, Yangyang Lu, Zhijun Wang, Shouling Wu

**Affiliations:** 1 Department of Cardiology, Affiliated Hospital of North China University of Science and Technology, Tangshan, China; 2 Department of Cardiology , Tangshan Worker’s Hospital, Tangshan, China; 3 Department of Respiratory and Critical Care Medicine, Cangzhou People’s Hospital, Cangzhou, China; 4 Department of Cardiology, KaiLuan General Hospital, Tangshan, China

**Keywords:** Sleep disorders, Triglyceride glucose index, HOMA-IR, NHANES

## Abstract

**Background:**

To determine the association between sleep disorders and Triglyceride glucose index.

**Methods:**

A cross-sectional analysis of the 2005 to 2008 National Health and Nutrition Examination Survey (NHANES) was performed. The 2005 to 2008 NHANES national household survey for adults ≥ 20 years was examined for the sleep disorders.TyG index: ln [triglyceride (mg/ dL) × fasting blood glucose (mg/dL)/2].Multivariable logistic and linear regression models were used to explore the association between the TyG index and sleep disorders.

**Results:**

A total of 4,029 patients were included. Higher TyG index is significantly associated with elevated sleep disorders in U.S. adults. TyG was moderately correlated with HOMA-IR (Spearman r = 0.51). TyG was associated with higher odds of sleep disorders(adjusted OR [aOR],1.896; 95% CI, 1.260 2.854), Sleep apnea (aOR, 1.559; 95% CI, 0.660 3.683), Insomnia(aOR, 1.914;95% CI, 0.531 6.896), and Restless legs (aOR, 7.759; 95% CI,1.446 41.634).

**Conclusions:**

In this study, our result shown that population with higher TyG index are significantly more likely to have sleep disorders in U.S. adults.

**Supplementary Information:**

The online version contains supplementary material available at 10.1186/s12888-022-04434-9.

## Introduction

Humans spend about one-third of their time sleeping, either to recover or to rest [1]. Sleep disorders are currently considered a public health disease by the Centers for Disease Control (CDC) [2].It cost approximately $3400 to $5200/person/year for health care [3]. Previous studies have shown that sleep disturbances are significantly associated with decreased quality of life and increased metabolic disease, arterial stiffness [4],cardiovascular disease, and mortality [5].

Several observational studies in large populations suggest that insulin resistance (IR) may be a major cause of sleep disturbance [6]. The triglyceride glucose index, which has been reported to be significantly associated with IR, is a simple and reliable surrogate indicator for IR [7–9].The association of Triglyceride glucose index (TyG) with cardiovascular outcomes and the onset of diabetes has been demonstrated [10–13].TyG may also be a risk factor for sleep disorders, and this study aimed to explore the relationship between TyG and sleep disorders.NHANES includes a nationally representative sample of U.S. adults. Therefore, to assess the association of TyG with sleep disorders, this study collected datasets from the National Health and Nutrition Examination Survey (NHANES).

## Methods

### Study population

This is a cross-sectional study with data from the National Health and Nutrition Examination Survey (2005–2008). The survey component included diet, questionnaires, and physiological measurements, as well as laboratory tests supervised by trained medical staff. In addition, NHANES employs various modern equipment to make data collection more reliable and efficient. In addition, each participant receives compensation and medical outcome reporting, which increases participant compliance. A total sample size of 20,497 adults was assessed from 2005–2008, Fig. 1 shows the study design and inclusion criteria, and participants who were excluded due to missing information on either covariate(missing data on sleep disorders,TyG index,HOMA-IR,fall asleep time,age, gender, race, smoke, drink,BMI,MVPA, Hypertension,Diabetes, CVD and cancer). The participants' medication information in the past month based on these modules, RXDUSE (Taken prescription medicine, past month),RXDDRUG(Generic drug name).Find the drug code via the RXQ_DRUG module.Only publicly available data were used in the analysis, and the NHANES protocol was approved by the National Center for Health Statistics (NCHS) Research Ethics Review Board approval.

**Fig. 1:**
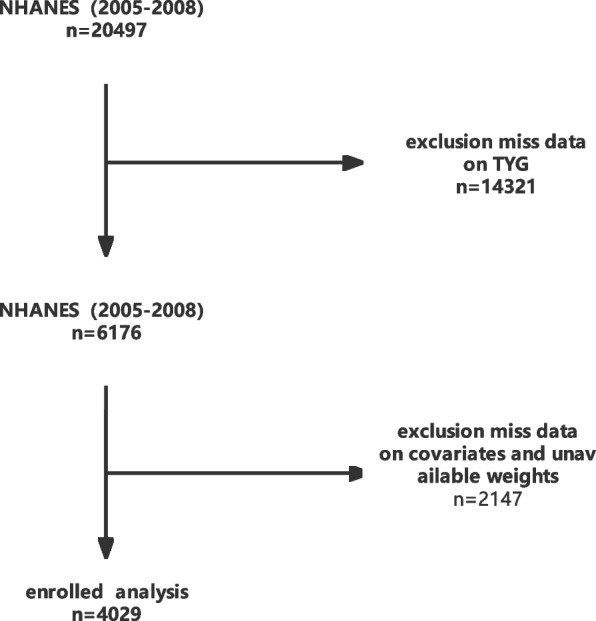
Flow chart of subject selection

### Data collection and definitions

TyG index was calculated as ln [fasting triglycerides (mg/dL) x fasting glucose (mg/dL)/2] [14]. HOMA-IR index was calculated as [fasting glucose (mmol/L) x fasting insulin (μU/mL)/22.5] [15]. Sleep disorders was self-reported by participants. For each adult, standard demographic data were collected. The sleep disorders module was queried for the NHANES question SLQ060, SLQ050: “Have you ever been told by a doctor or other health professional that you have a sleep disorder?”, “Have you ever told a doctor or other health professional that you have trouble sleeping?”,with those responding “yes” subsequently considered to have a sleep disorder in further analysis and SLQ070:People who self-report having Sleep Apnea,Insomnia,Restless Legs or other sleep disorders.,with those responding “yes” subsequently considered to have a sleep disorder in further analysis.

### Assessment of covariates

Age, gender, race (Mexican American, Other Hispanic, non-Hispanic white,non-Hispanic black and other race) were obtained by interviews and physical examinations. Existing smokers were defined as those who smoked 100 or more cigarettes and smoked at the time of the survey. Heavy alcohol consumption was defined as those who had consumed 12 or more glasses of alcohol/for life and who had consumed alcohol at the time of the survey.Leisure levels were calculated as the number of minutes per week during which participants reported participating in moderate to vigorous physical activity (MVPA).Participants with a BMI of 25 kg/m2 were considered overweight in accordance with the limit values.Pre-existing co-morbidities initially included a history of CVD, including coronary artery disease, angina pectoris, myocardial infarction and stroke (yes/no);diabetes (categorised as physician-diagnosed and undiagnosed diabetes); hypertension (categorised as diagnosed hypertension and no hypertension);cancer (categorised as diagnosed cancer and no cancer).

### Statistical analysis

Sample weights were used for analysis in order to account for complex survey design and non-response to NHANES.Continuous variables were summarized as mean 95%CI or median (interquartile range) depending on variable distribution, and categorical variables as count (proportion). TyG was compared with HOMA-IR by using Spearman correlation and with sleep disorders. Calculated the area under a receiver-operating characteristic curve generated without covariate adjustment.Multivariate logistic regression and linear analysis was then performed to assess the contribution of TyG index to sleep disorders,Model 1 was unadjusted. Model 2 was adjusted for age, gender, and race.Model 3 was adjusted for age,gender,race,BMI,smoke,drink,MVPA,Hypertension,Diabetes,CVD and cancer.The dose–response association was assessed on a continuous scale with restricted cubic spline curves. The subgroup variable stratified analysis was presented with a fully adjusted Model 3. The log-likelihood test was used to evaluate the interaction effects of the TyG index with subgroup variables.Sensitivity analysis was performed to evaluate which was not affected by taking hypotensive drugs, lipid-lowering drugs or hypoglycemic drugs.All statistical analyses were performed by using SAS version 9.4 (SAS Institute Inc., Cary, NC, USA) and R version 4.1.3 (R Core Team 2020) with a 2-sided *P* < 0.05 considered statistically significant.

## Results

### Baseline data for study participants

Figure 1 presented the study design, sampling and exclusion;and 16,468 participants were excluded due to missing information on either covariate(missing data on sleep disorders,TyG index,HOMA-IR,fall asleep time,age, gender, race, smoke, drink,BMI,MVPA, Hypertension,Diabetes, CVD and cancer).Our final sample included 4,029 NHANES participants, of which 48.61% were female and 71.76% were non-Hispanic whites (table.1). Population-weighted mean age was 46.9 years.Only 25.48% of people do physical activity every week.81.18% consumped at least 12 alcohol drinks/lifetime and drunk at the time of survey. About 50% who smoked at least 100 cigarettes and smoked at the time of survey. One-thirdof the population was hypertensive and obesity.

**Table 1:** Baseline and Study Measurements (*N* = 4,029)

Variable	value
Male%	1988(48.61)
Race%	
Mexican American	744(8.05)
Other Hispanic	315(4.12)
Non-Hispanic White	2018(71.76)
Non-Hispanic Black	798(10.70)
Other Race	154(5.38)
Smoke%	1923(48.54)
Drink%	3090(81.18)
MVPA%	882(25.48)
BMI ≥ 25%	1535(33.42)
Hypertension%	1434(31.57)
Diabetes%	552(9.70)
CVD%	342(6.45)
Cancer%	367(8.32)
Sleep Disorders%	959(24.98)
Sleep Apnea%	176(4.43)
Insomnia%	69(1.42)
Restless Legs%	14(0.28)
Age(year) mean 95%CI	46.69(45.61 47.77)
TyG index mean 95%CI	3.77(3.76 3.78)
logHOMA-IR mean 95%CI	0.78(0.73 0.83)
fall asleep time(minutes) mean 95%CI	21.24(20.28 22.19)

There were 959 (24.98%) persons with self-reported sleep disorders,4.43% of participants with sleep apnea,1.42% persons suffered from Insomnia.The prevalence of self-reported Restless Legs was 14(0.28%). Average time(mean 95%CI) tofall asleep costed was 21.24(20.28–22.19)minutes.

### Comparison of TyG to HOMA-IR and sleep disorders

**Fig. 2:**
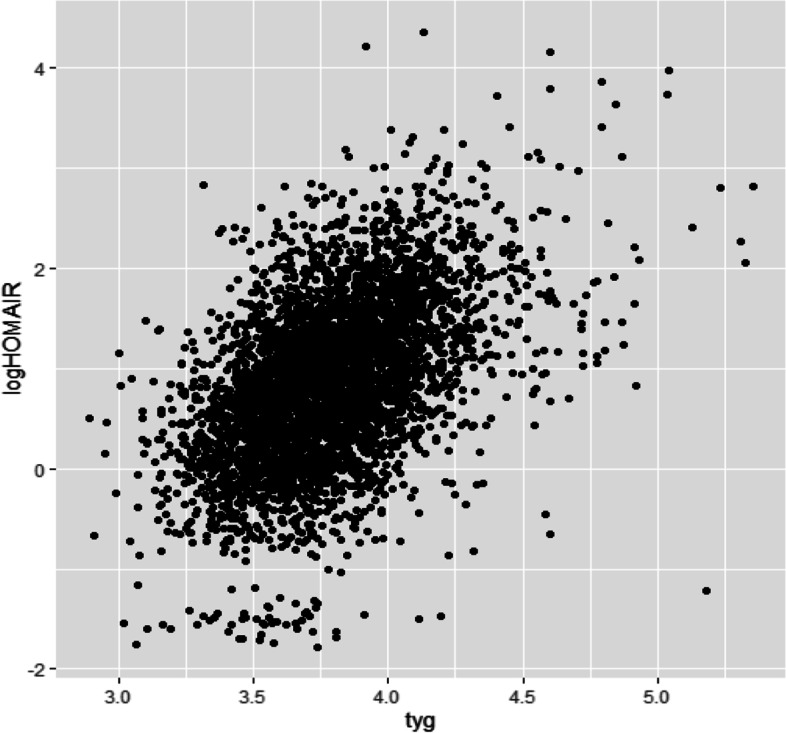
Scatterplot of triglyceride-glucose index against log-transformed homeostatic model assessment of insulin resistance (HOMA-IR). The population-weighted Spearman’s rho was 0.51

In the analyzed samples, TyG is Moderately correlated with HOMA-IR, corresponding to The population-weighted Spearman r was 0.51. Figure 2 Display participant-level TyG measurements HOMA-IR for log transformation.

**Fig. 3:**
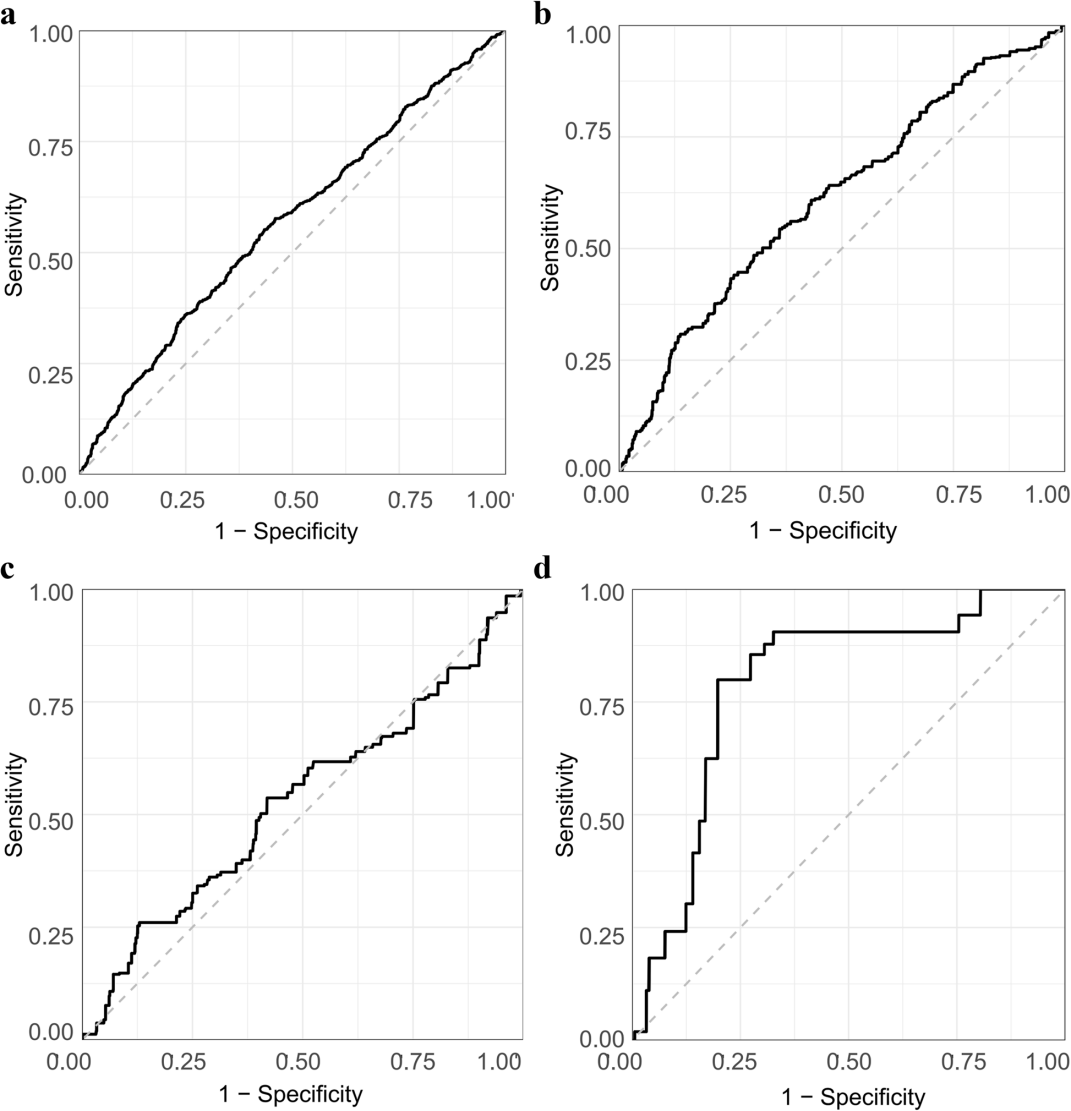
**a** ROC for TyG to Sleep disorders(AUC = 0.56). **b** ROC for TyG to Sleep apnea(AUC = 0.61). **c** ROC for TyG to Insomnia(AUC = 0.53). **d** ROC for TyG to Restless Legs(AUC = 0.79)

Figure 3 exhibited the population-weighted receiver-operating characteristic curve (ROC) of sleep disorders,sleep apnea,Insomnia and Restless Legs.The AUC for TyG to sleep disorders is 0.56, TyG was doing well Differentiation of Restless Legs(AUC = 0.79).

### Associations of TyG, HOMA-IR with study outcomes

**Fig. 4:**
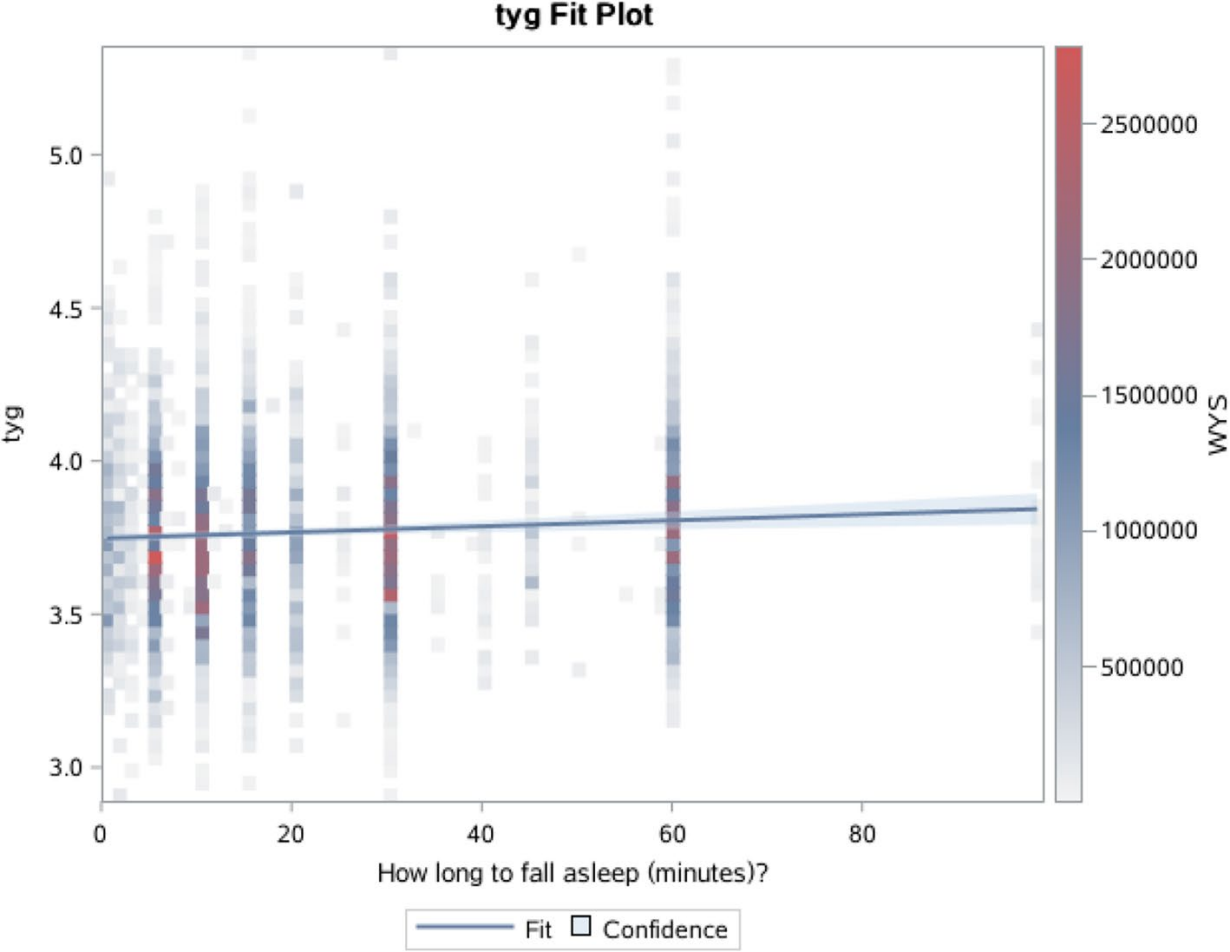
Association of triglyceride-glucose index (TyG) with time of fall asleep (minutes). The *p*-value indicates a test for linear trend with increasing number of symptoms derived by treating symptom count as a continuous variable

In Table 2, after adjusting for covariates, higher TyG index was associated with higher relative risks of sleep disorders(OR = 1.87; 95% confidence interval (CI) 1.26, 2.85) and Restless Legs(OR = 7.76; 95% confidence interval (CI) 1.45, 41.63). After adjusting for the same covariates, the HOMA-IR index had an increased risk of sleep disorders (OR = 1.21; 95% confidence interval (CI) 1.05, 1.39) and Sleep Apnea(OR = 2.01; 95% confidence interval (CI) 1.56, 2.59). Higher TyG means longer time to fall asleep(P value for linear trend, < 0.01)(Fig. 4).

**Table 2:** Association of triglyceride-glucose index (TyG) with study outcomes(*N* = 4,029)

Variable	TyG index	logHOMA-IR
Sleep disorders	1.896(1.260 2.854)	1.210(1.050 1.394)
Sleep apnea	1.559(0.660 3.683)	2.006(1.557 2.585)
Insomnia	1.914(0.531 6.896)	1.055(0.676 1.649)
Restless legs	7.759(1.446 41.634)	1.059(0.435 2.582)

adjusted for age,gender,race,BMI,smoke,drink,MVPA,Hypertension,Diabetes,CVD and cancer

**Fig. 5:**
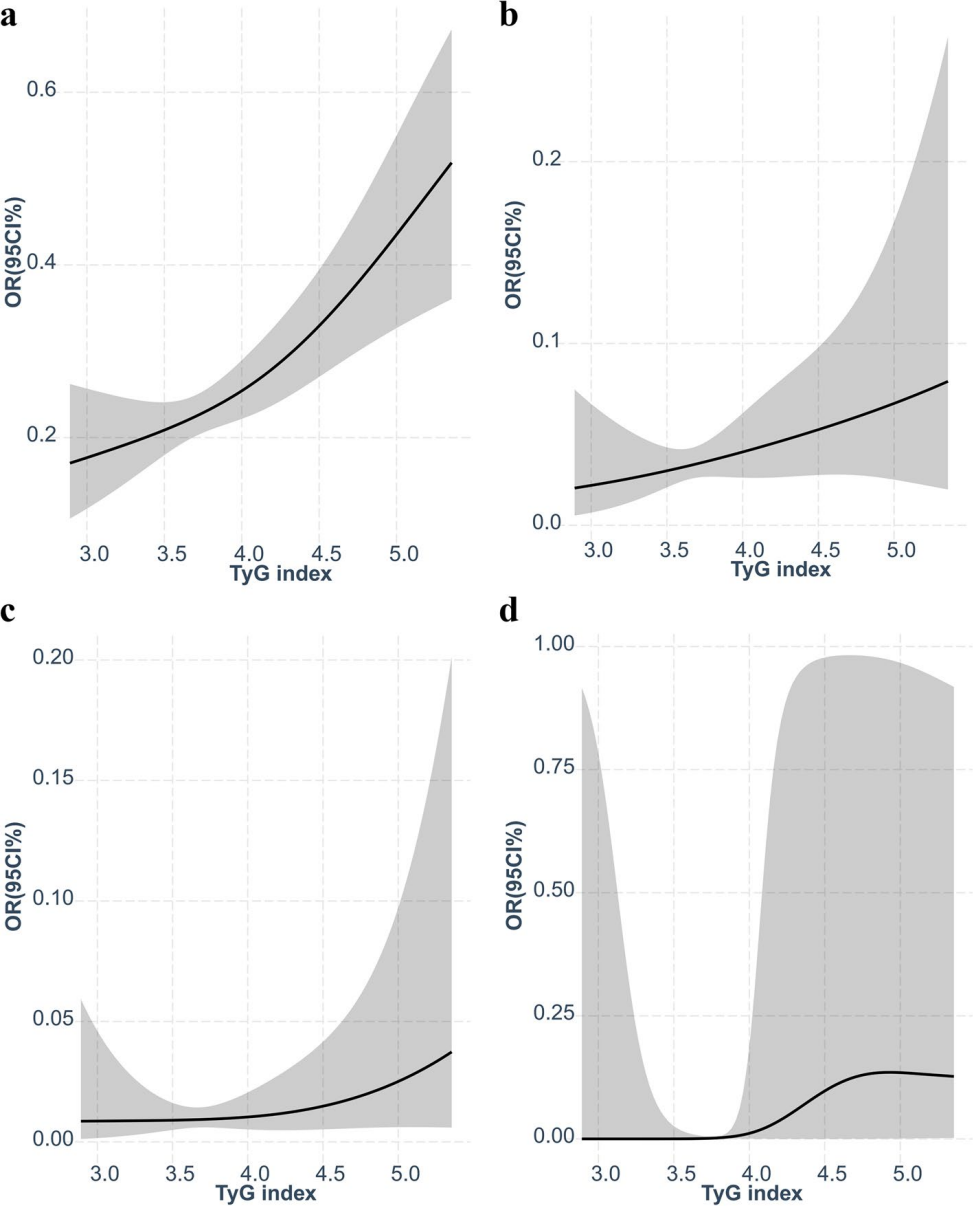
**a** Restricted cubic spline fitting for the association between TyG index levels with sleep disorders. **b** Restricted cubic spline fitting for the association between TyG index levels with sleep apnea. **c** Restricted cubic spline fitting for the association between TyG index levels with Insomnia. **d** Restricted cubic spline fitting for the association between TyG index levels with Restless Legs

As shown in Fig. 5, Restricted cubic spline curves suggested that the relationship between TyG index and sleep disorders,sleep apnea,insomnia and Restless Legs was linear. We analyzed the association of TyG with sleep disorders stratified by age(e_table.1), sex(e_table.2), and race(e_table.3), and we found no interaction effects, the confidence interval was too wide, which precluded meaningful inference.

Sensitivity analysis (e_table.4) was performed to evaluate which was not affected by taking lipid-lowering drugs,hypotensive drugs or hypoglycemic drugs.Since TyG appeared to be more strongly correlated with findings than HOMA-IR, we next tested whether the correlation of TyG with findings was independent of HOMA-IR. With additional adjustment for HOMA-IR,TyG continued to be associated with sleep disorders and Restless Legs.

## Discussion

In this nationally representative study, the relationship between TyG and sleep disorders was evaluated.The main finding of this paper is that TyG was associated with sleep disorders in the American adult population.This finding was also validated in adjusting the influence of HOMA-IR and was not affected by the use of lipid-lowering, hypotensive or hypoglycemic medications.We also found that increased exposure to TyG could increase the risk for Restless Legs, and the effect of HOMA-IR on Restless Legs was not significant,associations with Restless Legs seemed largely independent of IR (insulin resistance).

A previous study showed that participants with a higher TyG index had a higher risk of obstructive sleep apnea than the low-level group in Korean adult [16]. It may be related to differences in race or adjusted covariates,we found no positive effect of TyG index on sleep apnea in our study.However,we found the association between HOMA-IR and sleep apnea,similar to those previously published [17].After adjustment for HOMA-IR, changes in sleep disturbances suggesting insulin resistance did not fully explain the potential impact of TyG on the findings. Compared with the HOMA-IR metric, TyG is a lower-cost measure for IR [18, 19].In a large population.TyG index can serve as a practical alternative of IR measurement.In addition, the correlation between IR and sleep disturbances has already been documented [20].

Another significant finding was drawn from our linear trend analysis. This study shown that higher TyG means longer time to fall asleep,Edward etl found that longer periods to fall asleep (> 30 min) were associated with higher fasting insulin only for women [21], it confirmed our results.At the Circadian clocks level,several researchers have explored the interaction of gene behavior and have shown that interactions between diet and clock gene mutations affect fasting blood glucose [22], insulin resistance [23, 24], and T2DM [25].A study shows a correlation between improvements Insulin sensitivity and indicators of increased sleep duration After 40 days of sleep (approximately 45 min extra per night) [26],which partly explained our findings.Bosco D et al. found that IGT (prediabetes) is frequently associated with idiopathic RLS(Restless Legs Syndrome) [27].We also found that association between TyG index and Restless Legs with adjusted HOMA-IR.It suggests that prediction of TyG to sleep disorders is not only related to metabolism, but also genetics.In patients with dysglycemia Metabolic RLS may be due to simultaneous reduction in Inhibitory dopaminergic control of the dorsal horn of the spinal cord Excitatory nociceptive input due to peripheral Neuropathy [28].Basic research is still needed to complete the mechanisms of genetics.

Results from our study shown that a linear dose–response relationship between TyG and various sleep disorders, suggesting that regardless of their causal relationship, the earlier interventions on these two modifiable indicators, the greater the benefit.The American Heart Association proposed to take sleep health as one of Life’s Essential 8 [29], blood lipids and blood glucose are also included.Therefore, the combined improvement of sleep quality and TyG may improve cardiovascular health more than a single improvement,evidence from cohort studies is required.There were numerous cross-sectional and prospective epidemiological studies have shown that insufficient and perhaps excessive sleep time predisposes to systemic and central obesity, the metabolic syndrome, cardiovascular disease and all-cause mortality [5, 30].However,a recent prospective epidemiologic study reported for the first time that fasting hyperinsulinaemia and insulin resistance (as assessed by the HOMA-IR index) preceded incident ‘observed apnoeas’ over a 6-year follow-up period [31].Importantly, this link is bidirectional: on the one hand, the circadian clock regulates energy intake and metabolic pathways throughout the organism, while on the other hand feeding behavior and the nutrient composition of the diet influence the circadian clock itself, especially peripheral metabolic organs and their outputs [4].Our study shows a positive relationship between TyG index and sleep disturbance, but the mechanism of the interaction between metabolism and sleep disturbance remains to be investigated.

Our study was the first to investigate the relationship between TyG and sleep disorders, and the positive relationship has been observed. In this study, the sleep disorders includes several kinds of sleep problems, which could largely reflect people's state of sleep. the findings would serve as a reminder to the public to pay more attention to sleep health.This study provides a foundation for future multi-centre cohort studies on sleep disorders and TyG index.Previous studies showed insulin resistance were associated with sleep disorders [6], which may partly explain the significant relationship of TyG index with sleep disorders.However, our investigation has limitation. it was a study by observation, and causality cannot be demonstrated.

## Conclusions

After adjusting for case complexity, a high TyG index was associated with higher odds of individuals with sleep disorders in the general population. Insulin resistance did not fully explain the findings.Our finding suggests that the TyG index may be an independent predictor of the development of sleep disorders.

## Supplementary Information

**Additional file 1:** **e_table.1.** Association of triglyceride-glucose index (TyG) with study outcomes, stratified by age.

**Additional file 2:** **e_table.2.** Association of triglyceride-glucose index (TyG) with study outcomes, stratified by gender.

**Additional file 3:** **e_table.3.** Association of triglyceride-glucose index (TyG) with study outcomes, stratified by race

**Additional file 4:** **e_table.4.** Association of triglyceride-glucose index (TyG) with study outcomes

## Data Availability

All data generated or analyzed during this study is included at this URL. https://www.cdc.gov/nchs/nhanes/index.htm.

## Abbreviations

NHANE: National health and nutrition examination survey
TyG: Triglyceride glucose index
MVPA: Moderate to vigorous-intensity physical activity
CVD: Cardiovascular disease
BMI: Body mass index
HOMA-IR: Homeostasis model assessment of insulin resistance
RLS: Restless legs syndrome
