# Supplementary material for: Association between triglyceride glucose index and sleep disorders: results from the NHANES 2005–2008
Source: BMC Psychiatry. 2023 Mar 10;23:156. doi: 10.1186/s12888-022-04434-9 (PMC10007799; doi:10.1186/s12888-022-04434-9)
Supplement: Supplementary file 1 — Additional file 1: e_table.1. Association of triglyceride-glucose index (TyG) with study outcomes, stratified by age. [file 12888_2022_4434_MOESM1_ESM.docx]

e_table.1 Association of triglyceride-glucose index (TyG) with study outcomes, stratified by age

| **Variable** | **Age** | | ***p*** for interaction |
| --- | --- | --- | --- |
|  | **age>60** | **age≤60** |  |
| Sleep disorders | 1.548(0.864 2.773) | 1.947(1.217 3.112) | 0.1335 |
| Sleep Apnea | 2.423(0.729 8.054) | 1.176(0.361 3.828) | 0.8629 |
| Insomnia | 4.864(0.982 24.079) | 1.429(0.351 5.822) | 0.1781 |
| Restless Legs | 33.921(4.512 255.013) | 4.593(0.276 76.533) | 0.4438 |

adjusted for gender,race,BMI,smoke,drink,MVPA,Hypertension,Diabetes,CVD and cancer.
