# Supplementary material for: Association between triglyceride glucose index and sleep disorders: results from the NHANES 2005–2008
Source: BMC Psychiatry. 2023 Mar 10;23:156. doi: 10.1186/s12888-022-04434-9 (PMC10007799; doi:10.1186/s12888-022-04434-9)
Supplement: Supplementary file 2 — Additional file 2: e_table.2. Association of triglyceride-glucose index (TyG) with study outcomes, stratified by gender. [file 12888_2022_4434_MOESM2_ESM.docx]

e_table.2 Association of triglyceride-glucose index (TyG) with study outcomes, stratified by gender

| **Variable** | **Gender** | | ***p* for interaction** |
| --- | --- | --- | --- |
|  | **male** | **female** |  |
| Sleep disorders | 1.832(1.048 3.204) | 2.039(1.055 3.941) | 0.9121 |
| Sleep Apnea | 3.325(0.700 15.798) | 0.954(0.441 2.063) | 0.1535 |
| Insomnia | 0.976(0.165 5.781) | 6.540(1.419 30.142) | 0.2668 |
| Restless Legs | 7.022(0.540 91.291) | 12.404(6.227 24.708) | 0.3732 |

adjusted for age,race,BMI,smoke,drink,MVPA,Hypertension,Diabetes,CVD and cancer.
