# Supplementary material for: Association between triglyceride glucose index and sleep disorders: results from the NHANES 2005–2008
Source: BMC Psychiatry. 2023 Mar 10;23:156. doi: 10.1186/s12888-022-04434-9 (PMC10007799; doi:10.1186/s12888-022-04434-9)
Supplement: Supplementary file 3 — Additional file 3: e_table.3. Association of triglyceride-glucose index (TyG) with study outcomes, stratified by race [file 12888_2022_4434_MOESM3_ESM.docx]

e_table.3 Association of triglyceride-glucose index (TyG) with study outcomes, stratified by race

| **Variable** | **Race** | | | ***p* for interaction** |
| --- | --- | --- | --- | --- |
|  | **white** | **black** | **Hispanic** |  |
| Sleep disorders | 2.096(1.278 3.436) | 1.723(0.922 3.217) | 0.983(0.332 2.907) | 0.4804 |
| Sleep Apnea | 1.849(0.689 4.964) | 0.733(0.282 1.902) | 0.317(0.018 5.627 ) | 0.6126 |
| Insomnia | 1.726(0.378 7.891 ) | 1.443(0.291 7.155 ) | 1.685(0.145 19.592) | 0.5424 |
| Restless Legs | 6.537(0.699 61.110) | 667.993 (4.704 >999.999) | 749.241(0.972 >999.999) | 0.4108 |

adjusted for age,gender,BMI,smoke,drink,MVPA,Hypertension,Diabetes,CVD and cancer.
