# Supplementary material for: Association between triglyceride glucose index and sleep disorders: results from the NHANES 2005–2008
Source: BMC Psychiatry. 2023 Mar 10;23:156. doi: 10.1186/s12888-022-04434-9 (PMC10007799; doi:10.1186/s12888-022-04434-9)
Supplement: Supplementary file 4 — Additional file 4: e_table.4. Association of triglyceride-glucose index (TyG) with study outcomes [file 12888_2022_4434_MOESM4_ESM.docx]

e_table.4 Association of triglyceride-glucose index (TyG) with study outcomes

| **Variable** | **TyG index*** | **TyG index+** |
| --- | --- | --- |
| Sleep disorders | 1.647(1.049 2.588) | 1.911(1.204 3.032) |
| Sleep Apnea | 0.578(0.194 1.717) | 1.706(0.682 4.270) |
| Insomnia | 2.045(0.525 7.960) | 1.752(0.466 6.581) |
| Restless Legs | 10.141(1.057 97.338) | 12.457(3.098 50.086) |

adjusted for age,gender,race,BMI,smoke,drink,MVPA,Hypertension,Diabetes,CVD and cancer.

*additional adjustment for HOMA-IR.

+exclude by taking lipid-lowering drugs ,hypotensive drugs or hypoglycemic drugs.
